# Supplementary material for: Plasmodium Niemann-Pick type C1-related protein is a druggable target required for parasite membrane homeostasis
Source: eLife. 2019 Mar 19;8:e40529. doi: 10.7554/eLife.40529 (PMC6424564; doi:10.7554/eLife.40529)
Supplement: Figure 1—source data 1. [file elife-40529-fig1-data1.docx]

| **Compound** | **EC50 (nM)** | **Standard Error** | **Replicates** |
| --- | --- | --- | --- |
| MMV009108 | 454 | 40 | N=11 |
| MMV019662 | 605 | 131 | N=4 |
| MMV028038 | 500 | 98 | N=6 |

Potencies of compounds against parental (wild-type) parasites in Figure 1D)-F).
