# Supplementary material for: Plasmodium Niemann-Pick type C1-related protein is a druggable target required for parasite membrane homeostasis
Source: eLife. 2019 Mar 19;8:e40529. doi: 10.7554/eLife.40529 (PMC6424564; doi:10.7554/eLife.40529)
Supplement: Figure 1—source data 2. [file elife-40529-fig1-data2.docx]

| **Clone** | **Compound** | **EC_50_ fold change**  **compared to parent** | **Standard error** | **Biological**  **Replicates** | **Paired**  **T-test**  **P-value** | **Figure** |
| --- | --- | --- | --- | --- | --- | --- |
| A1108A | 009108 | 0.99 | 0.1 | 5 | 0.9 | 1D |
| A1108T | 009108 | 3.6 | 0.4 | 5 | 0.005 | 1D |
| A1208E | 028038 | 15 | 4 | 4 | 0.03 | 1E |
| F1436I | 019662 | 22 | 6 | 4 | 0.04 | 1F |
| A1108T | 028038 | 7.1 | 1 | 4 | 0.007 | 1-S3A |
| A1108T | 019662 | 4.9 | 0.4 | 4 | 0.003 | 1-S3B |
| F1436I | 009108 | 7.6 | 2 | 4 | 0.06 | 1-S3C |
| F1436I | 028038 | 5.6 | 1 | 4 | 0.04 | 1-S3D |
| A1208E | 009108 | 1.2 | 0.2 | 4 | 0.4 | 1-S3E |
| A1208E | 019662 | 1.9 | 0.1 | 4 | 0.004 | 1-S3F |

Resistance of allelic-exchange modified parasites, compared to parental parasites.
