# Supplementary material for: Plasmodium Niemann-Pick type C1-related protein is a druggable target required for parasite membrane homeostasis
Source: eLife. 2019 Mar 19;8:e40529. doi: 10.7554/eLife.40529 (PMC6424564; doi:10.7554/eLife.40529)
Supplement: Figure 2—source data 1. [file elife-40529-fig2-data1.docx]

| **Compound** | **EC_50_ fold change**  **under knockdown** | **Standard error** | **Biological**  **Replicates** | **Paired T-test**  **P-value** | **Figure** |
| --- | --- | --- | --- | --- | --- |
| 009108 | 18 | 1.4 | 3 | 0.007 | 2E |
| 028038 | 70 | 2.3 | 2 | 0.02 | 2F |
| 019662 | 35 | 0.56 | 2 | 0.01 | 2G |
| MFQ | 0.76 | 0.015 | 2 | 0.04 | 2H |

Shifts in EC_50_s under PfNCR1 knockdown.
