# Supplementary material for: Plasmodium Niemann-Pick type C1-related protein is a druggable target required for parasite membrane homeostasis
Source: eLife. 2019 Mar 19;8:e40529. doi: 10.7554/eLife.40529 (PMC6424564; doi:10.7554/eLife.40529)
Supplement: Figure 4—source data 1. [file elife-40529-fig4-data1.docx]

EC_50_ fold change to KAE609 under PfNCR1 knockdown.

| **EC_50_ fold change**  **under knockdown** | **Standard error** | **Biological**  **Replicates** | **Paired T-Test P-Value** | **Figure** |
| --- | --- | --- | --- | --- |
| 1.2 | 0.15 | 3 | 0.3 | Fig 4 - SC |
