# Supplementary material for: Plasmodium Niemann-Pick type C1-related protein is a druggable target required for parasite membrane homeostasis
Source: eLife. 2019 Mar 19;8:e40529. doi: 10.7554/eLife.40529 (PMC6424564; doi:10.7554/eLife.40529)
Supplement: Supplementary file 1. [file elife-40529-supp1.docx]

**Primers used in this study**

| **Primer name** | **Purpose** | **Sequence (5’-3’)**  **For mutation constructs, mutations are in bold letters** |
| --- | --- | --- |
| AR1-F | Allelic exchange | CACTATAGAACTCGAGCCATCAAAAATTGTATCTATGGAAG |
| AR1-R | Allelic exchange | CTGCACCTGGCCTAGGATGTAGTGGGCCAAAACTGGAAAGAAG |
| Mut-1 | Mutation | GTCTATATATGGATGTACT**CTC**ATGAAGAAAGGAATAAAATATGATAAA**A**CATTTCCTGTAGATTCATATG |
| Mut-2 | Mutation | GCATAATACATTAGAATCACAAGAATT**C**GTTACATCAGTAG**A**AAATGGTTTTACATTTTTTTTAAATAAAAAT |
| Mut-3 | Mutation | GTACCTTATTTTTTTCAAATAAAGAT**A**TTACAGTCATATTTTTCCAAACATTAAGC |
| Probe1 | Southern blot | TCATTAAAGTACATCCATATATAGAC |
| Probe2 | Southern blot | GACCATCAAGTGATGAAGAATATAAAG |
| RHR1 | Aptamer-tagging | cataaatattgtgagtgttcatccttaagaaaagaaaacccacaagaatttta |
| RHR2 | Aptamer-tagging | tacgtcataaggatagacgtcGACGTCATGTAGTGGGCCAAAAGAACTAAGAAGTACTGGCAAGAAC |
| LHR1 | Aptamer-tagging | caatggcccctttccgGGCGCGCCgaacaaattaaaataaatgattttg |
| LHR2 | Aptamer-tagging | taaaattcttgtgggttttcttttcttaaggatgaacactcacaatatttatg |
| GRNA1 | PfNCR1 gRNA | TAAGTATATAATATTTTAATGTAGTGGGCCAAAACGTTTTAGAGCTAGAA |
| GRNA2 | PfNCR1  gRNA | TTCTAGCTCTAAAACGTTTTGGCCCACTACATTAAAATATTATATACTTA |
| Int1 | PCR of *pfncr1* locus | GTTACATCAGTAGCAAATGG |
| Int2 | PCR of *pfncr1* locus | ggctgttcgtgccttttgtaagag |
| Int3 | PCR of *pfncr1* locus | GATTATGAAAATATATCCATATATGAATGG |
| Int4  Int5  Int6  GFP1 | PCR of pfncr1  locus  control PCR  control PCR  Reverse in GFP for sequencing | ATGTAGTGGGCCAAAACTGGAAAGAAG  CTACCGAATTAGCTACTAAGGATAC  GTAAATTTGTATTTTCCCAATGTAC  GAAAAGTTCTTCTCCTTTACTCAT |
| GFP2  Comp1 | Reverse in GFP for PCR  Cloning gene | CAGAAAATTTGTGCCCATTAACATCAC  ACGATTTTTTCTCGAGATGTTCGTAAAAAATTTTATACATAAA |
| Comp2 | Cloning gene | CTGCACCTGGCCTAGGATGTAGTGGGCCAAAACTGGAAAGAAG |
| gBlock1 | SERA5-gBlock for split-GFP | ATGAAGAGTTATATTTCGCTCTTCTTTATCTTGTGCGTCATCTTCAATAA  GAATGTCATCAAATGTACTGGCGAGAGTATGAGCAAAGGAGAAGAACT  TTTCACTGGAGTTGTCCCAATTCTTGTTGAATTAGATGGTGATGTTAAT  GGGCACAAATTTTCTGTCAGAGGAGAGGGTGAAGGTGATGCTACAAT  CGGAAAACTCACCCTTAAATTTATTTGCACTACTGGAAAACTACCTGTT  CCATGGCCAACACTTGTCACTACTCTGACCTATGGTGTTCAATGCTTTT  CCCGTTATCCGGATCACATGAAAAGGCATGACTTTTTCAAGAGTGCCA  TGCCCGAAGGTTATGTACAGGAACGCACTATATCTTTCAAAGATGACG  GGAAATACAAGACGCGTGCTGTAGTCAAGTTTGAAGGTGATACCCTTG  TTAATCGTATCGAGTTAAAGGGTACTGATTTTAAAGAAGATGGAAACAT  TCTCGGACACAAACTCGAGTACAACTTTAACTCACACAATGTATACATC  ACGGCAGACAAACAAAAGAATGGAATCAAAGCTAACTTCACAGTTCGC  CACAACGTTGAAGATGGTTCCGTTCAACTAGCAGACCATTATCAACAAA  ATACTCCAATTGGCGATGGCCCTGTCCTTTTACCAGACAACCATTACCT  GTCGACACAAACTGTCCTTTCGAAAGATCCCAACGAAAAGTAA |
| gBlock2 | 3XHA-GFP11 | CCTAGGTACCCGTACGACGTCCCGGACTACGCTGGCTATCCCTATGAT  GTGCCCGATTATGCGTATCCTTACGATGTTCCAGATTATGCCGATGGA  GGGTCTGGTGGCGGATCAACAAGTCGTGACCACATGGTCCTTCATGA  GTACGTAAATGCTGCTGGGATTACATAACGGCCG |
| GFP1-10-1F | SERA5-  GFP1-10F | AAATATATCAcctaggATGAAGAGTTATATTTCGCTCTTCTTTATCTTG  TGC |
| GFP1-10-1R | GFP1-10R | ATAACTCGACcttaagTTACTTTTCGTTGGGATCTTTCGAAAGGACAG |
| GFP1-10-  2F | GFP1-10F | AAATATATCAcctaggATGAGCAAAGGAGAAGAACTTTTC |
| GFP11-F | PfNCR1-GFP11 | ggacgtcgtacgggtaCCTAGGATGTAGTGGGCCAAAAGAACTAAGAA  GTACTGGC |
| GFP11-R | PfNCR1-GFP11 | taggtgacactatagaaCTCGAGGAACAAATTAAAATAAATGATTTTG |
| Plasmid1 | Tag integration screening | GTAGACCCCATTGTGAGTAC |
| eGFP-F | PCR of eGFP | gaaaagtgccacctgacgtcCTCTGAGCTTCTTCTTTGTTAACC |
| eGFP-R | PCR of eGFP | tatataactcgacgcggccgTTACTTGTACAGCTCGTCCATGCCG |
| Rh3-5’-F | Cloning eGFP into Rh3 | TATTTCATATGCGTGATTTATATGATTTAGcttaagGTGCTAAAGAGAGTA  ATGTTTCTG |
| Rh3-5’-R | Cloning eGFP into Rh3 | GGAGACCGGCagatctCGCCCATTTATTTCGTCATATG |
| Rh3-3’-F | Cloning eGFP into Rh3 | TTTCCTTATAagatctGCAATAAATGTGGTTTTAGAAGAATTAG |
| Rh3-3’-R | Cloning eGFP into Rh3 | CAGAAACATTACTCTCTTTAGCACcttaagCTAAATCATATAAATCACGCA  TATGAAATA |
| Rh3-G1 | Rh3 gRNA | TAAGTATATAATATTtggtaatacagaaatggatgGTTTTAGAGCTAGAA |
| Rh3-G2 | Rh3 gRNA | TTCTAGCTCTAAAACcatccatttctgtattaccaAATATTATATACTTA |
